# The Association Between Cortisol Response to Mental Stress and High-Sensitivity Cardiac Troponin T Plasma Concentration in Healthy Adults

**DOI:** 10.1016/j.jacc.2013.05.070

**Published:** 2013-10-29

**Authors:** Antonio I. Lazzarino, Mark Hamer, David Gaze, Paul Collinson, Andrew Steptoe

**Affiliations:** ∗ Department of Epidemiology and Public Health, University College London, London, United Kingdom; † Chemical Pathology, Clinical Blood Sciences, St. George's Healthcare NHS Trust, London, United Kingdom

**Keywords:** atherosclerotic plaque, computed tomography, myocardial infarction, psychological stress, troponin T, AMI, acute myocardial infarction, BMI, body mass index, CAC, coronary artery calcification, CRP, C-reactive protein, CVD, cardiovascular disease, hs-cTnT, high-sensitivity cardiac troponin T, IL, interleukin, HDL, high-density lipoprotein, LDL, low-density lipoprotein

## Abstract

**Objectives:**

The objective of this study was to examine the association between cortisol response to mental stress and high-sensitivity cardiac troponin T (hs-cTnT) in healthy older individuals without history of cardiovascular disease (CVD).

**Background:**

Mental stress is a recognized risk factor for CVD, although the mechanisms remain unclear. Cortisol, a key stress hormone, is associated with coronary atherosclerosis and may accentuate structural and functional cardiac disease.

**Methods:**

This cross-sectional study involved 508 disease-free men and women aged 53 to 76 years drawn from the Whitehall II epidemiological cohort. We evaluated salivary cortisol response to standardized mental stress tests (exposure) and hs-cTnT plasma concentration using a high-sensitivity assay (outcome). We measured coronary calcification using electron-beam dual-source computed tomography and Agatston scores.

**Results:**

After adjustment for demographic and clinical variables associated with CVD as well as for inflammatory factors, we found a robust association between cortisol response and detectable hs-cTnT (odds ratio [OR]: 3.98; 95% confidence interval [CI]: 1.60 to 9.92; p = 0.003). The association remained when we restricted the analysis to participants without coronary calcification (n = 222; OR: 4.77; 95% CI: 1.22 to 18.72; p = 0.025) or when we further adjusted for coronary calcification in participants with positive Agatston scores (n = 286; OR: 7.39; 95% CI: 2.22 to 26.24; p = 0.001).

**Conclusions:**

We found that heightened cortisol response to mental stress was associated with detectable plasma levels of cTnT using high-sensitivity assays in healthy participants, independently of coronary atherosclerosis. Further research is needed to understand the role of psychosocial stress in the pathophysiology of cardiac cell damage.

Mental stress is becoming increasingly recognized as a risk factor and trigger for cardiovascular disease (CVD) events 1, 2, 3. Stress can be studied in several ways, including epidemiological studies, laboratory-based psychophysiological testing, and animal research. Psychophysiological testing allows mechanisms to be studied by measuring biological responses to standardized behavioral challenge. Stress markers relevant to CVD include proinflammatory factors, cortisol level, heart rate variability, and hemostatic processes 4, 5. Mental stress initiates the release of cortisol by activating corticotropin-releasing factor and arginine vasopressin neurons in the paraventricular nucleus of the hypothalamus (6). This leads to the release of adrenocorticotropic hormone from the pituitary gland, which triggers release of glucocorticoids from the adrenal glands. Cortisol has attracted relatively little attention as a mechanism linking stress and CVD. However, several population studies have demonstrated associations between diurnal cortisol patterns and subclinical atherosclerosis 7, 8. Additionally, a flatter slope in the decline of cortisol levels across the day (thought to be a marker of chronic stress) is associated with an increased risk of CVD mortality in British civil servants (9); 24-h urinary cortisol level was associated with CVD death in the InCHIANTI (Invecchiare [Aging] in Chianti [region in Italy]) prospective cohort study of older people (10); serum cortisol levels were found to be a cardiac event risk predictor in patients with chronic heart failure; and cardiac event prediction based on cortisol levels was influenced by oxidative stress (11). Recent data from our laboratory have shown that heightened increases in salivary cortisol levels following standardized mental stress tests in healthy older individuals were associated with greater coronary artery calcification (CAC) and with CAC progression over 3 years 12, 13.

Cardiac troponin T (cTnT) is a plasma protein routinely used for the diagnosis of acute myocardial infarction (AMI) 14, 15. In clinical settings cTnT is measured using standard assays that have a detection limit of 10 ng/l (16) and a diagnostic threshold of 35 ng/l 14, 15. However, high-sensitivity assays (hs-cTnT) have now been developed with a lower detection limit of 3 ng/l 17, 18, 19. In healthy people not fulfilling any diagnostic criteria for AMI, greater hs-cTnT level is associated with a greater incidence of structural and functional heart disease, cardiovascular mortality, and all-cause mortality 20, 21. Among patients undergoing noncardiac surgery, the post-operative increase in hs-cTnT plasma concentration was associated with increased 30-day mortality (22). In a study of community-derived perimenopausal women, hs-cTnT level was associated with long-term mortality, independently of amino-terminal pro–B-type natriuretic peptide and other risk factors (23).

Cortisol is associated with coronary atherosclerosis, although whether this hormone plays a role in structural and functional cardiac disease remains unclear. The aim of our study was therefore to provide further insight into the role of cortisol in CVD by examining the association between cortisol responses to mental stress and hs-cTnT concentrations in healthy older individuals without a history of CVD, taking into account underlying coronary atherosclerosis. We hypothesized that high cortisol responders are individuals who are hyperreactive to mental stress. If these responses are elicited on a regular basis over many years, they might lead to chronic elevation in hs-cTnT concentration.

## Methods

### Study design

Our study involved participants drawn from the Whitehall II epidemiological cohort (24) for psychophysiological testing between 2006 and 2008. The criteria for entry into the study included no history or objective signs of clinical or subclinical CVD and no previous diagnosis or treatment for hypertension, inflammatory diseases, allergies, or kidney disease. CVD was defined as prior MI, stable or unstable angina, revascularization procedure, heart failure, transient ischemic attack, stroke, or electrocardiographic abnormalities (resting 12-lead electrocardiograms were taken). This information was confirmed by a telephone interview and verified from clinical data collected from the previous 7 phases of the Whitehall II study. Volunteers were of white European origin, aged 53 to 76 years; 56.5% were in full-time employment. Selection was stratified by grade of employment (current or most recent) to include higher and lower socioeconomic status participants. From the initially invited participants (N = 1,169), 27.6% were not eligible (mainly because of prescribed medications) and 25.9% declined to take part. Participants were prohibited from using any antihistamine or anti-inflammatory medication for 7 days before testing and were rescheduled if they reported colds or other infections on the day of testing. Participants gave full informed consent to participate in the study, and ethical approval was obtained from the University College London Hospitals Committee on the Ethics of Human Research.

### Data collection

#### Psychophysiological Testing

We carried out psychophysiological stress testing in either the morning or afternoon in a light temperature-controlled laboratory. This procedure was based on a protocol previously used in this laboratory (25). Participants were instructed to refrain from drinking caffeinated beverages and smoking for at least 2 h before the study and not to have performed vigorous physical activity or consumed alcohol the previous evening. After a 30-min rest period, baseline blood pressure (using an automated UA-779 digital monitor [A&D Instruments Ltd., Oxford, United Kingdom]) and a saliva sample were taken. Two behavioral tasks, designed to induce mental stress, were then administered in random order. The tasks were a computerized version of the Stroop task and mirror tracing, both of which have been used extensively in psychophysiological research (26). The tasks each lasted for 5 min. Saliva samples were collected immediately before and after the tasks for the assessment of salivary cortisol. The samples were collected using Salivettes (Sarsted, Leicester, United Kingdom), which were stored at −30°C until analysis. Levels of cortisol were assessed using a time-resolved immunoassay with fluorescence detection at the University of Dresden (Dresden, Germany). The intra-assay and interassay coefficients of variation (CVs) were less than 8%.

#### Cardiac Troponin T

Nonfasting blood samples were collected in EDTA tubes and centrifuged immediately at 2,500 rpm for 10 min at room temperature. Plasma was removed from the tube and aliquoted into 0.5-ml portions and stored at −80°C until analysis. We measured cTnT concentrations 75 min after the end of the mental stress test using a highly sensitive assay on an automated platform (Elecsys-2010 Troponin T hs STAT, Roche Diagnostics, Haywards Heath, United Kingdom), with a lower detection limit of 3 ng/l and a reported 99th percentile value in apparently healthy individuals of 13.5 ng/l, at which the CV is 9%, confirmed by in-house studies 17, 18, 19.

#### Covariates

We assayed baseline plasma interleukin (IL)-6 using a Quantikine high-sensitivity 2-site enzyme-linked immunosorbent assay (ELISA) from R&D Systems (Oxford, United Kingdom). The sensitivity of the assay ranged from 0.016 to 0.110 pg/ml, with intra-assay and interassay CVs of 7.3% and 7.7%, respectively. Baseline C-reactive protein (CRP) level was measured using a high-sensitivity ELISA (R&D Systems).

CAC was assessed with electron-beam computed tomography (Imatron C-150, General Electric, San Francisco, California) as previously described (27). In brief, 40 contiguous 3-mm slices were obtained during a single breath-hold starting at the carina and proceeding to the level of the diaphragm. Scan time was 100 ms/slice, synchronized to 40% of the R-R interval. Agatston and volumetric calcium scores were calculated to quantify the extent of CAC by a single experienced investigator blinded to the psychophysiological and clinical data on an Aquarius workstation (TeraRecon Inc., San Mateo, California). Because calcified volume was highly correlated with Agatston score (Spearman rho 0.99), we present data for Agatston score only.

Participants reported current smoking levels, weekly alcohol intake (units per week), employment grade (as a marker of social position), and hours of moderate or vigorous physical activity per week. We measured height and weight in light clothing for calculation of body mass index (BMI). Fasting blood samples were taken during a separate clinical assessment. Total and high-density lipoprotein (HDL) cholesterol and triglycerides were measured within 72 h in serum stored at 4°C using enzymatic colorimetric methods (28). Low-density lipoprotein (LDL) cholesterol level was derived using the Friedewald equation (29). Glucose homeostasis was assessed from glycosylated hemoglobin (HbA_1C_) concentration, assayed using boronate affinity chromatography, a combination of boronate affinity and liquid chromatography.

### Data analysis

We quantified the cortisol response to stress by subtracting the values of salivary cortisol concentration measured immediately after the behavioral tasks from the baseline. The resulting measure was normally distributed, and it was transformed for the main analyses into a binomial variable using a cut point at the value of 4 nmol/l, which corresponds to the mean value (0.54 nmol/l) + 1 SD (3.47 nmol/l). hs-cTnT was highly right skewed and was undetectable for 83.3% of the sample (below the lower detection limit of 3 ng/l); therefore, it was transformed into a binomial variable (detectable vs. undetectable). Agatston score (CAC) had a right-skewed distribution and was transformed for some analyses into a binomial variable by cutting at the value of zero (0 vs. >0) or 100 (<100 vs. ≥100). This threshold was based on the St. Francis Heart Study, which demonstrated maximum sensitivity and specificity for detecting cardiovascular events at a threshold calcium score ≥100 (30).

We described the study sample according to the exposure (salivary cortisol response to stress tasks) and the outcome (plasma detectable hs-cTnT) variables. Triglyceride and CRP levels had right-skewed distributions and were described using medians and interquartile ranges. Afterward we used multiple logistic regression to model the association between cortisol stress response and odds of detectable hs-cTnT. Cortisol responses may differ according to baseline cortisol levels and time of testing; therefore, these parameters were included as covariates. We also adjusted for age, sex, employment grade, and smoking because they are related to CVD and may confound the association between cortisol reactivity and CVD. Additionally, we took into account clinical variables that are known to be linked to CVD such as systolic blood pressure, total cholesterol/HDL ratio, and HbA_1c_. Moreover, we adjusted for CRP and IL-6 levels to account for vascular inflammation. Finally, we examined whether the association between cortisol reactivity and hs-cTnT was independent of underlying coronary atherosclerosis using several approaches: we adjusted for CAC score as a binomial variable using both cut points separately (0 and 100), carried out a subanalysis of participants with no detectable coronary calcification, and carried out a subanalysis of participants with positive CAC scores and in this case further adjusted for CAC score as a log-linear variable.

We carried out sensitivity analyses on cortisol responses by testing several different methods of quantification. To test for dose-response associations, we analyzed the cortisol response as a continuous variable; we tested a different cutoff for binomial analysis, defining positive responses as +0.5 SD instead of 1 SD; we calculated cortisol responses as the ratio between the post-stress and baseline values and tested the resulting variable as a linear variable and in binary analyses using both +1 SD and +0.5 SD as cut points. Additionally, we checked for an association between the baseline levels of salivary cortisol and hs-cTnT using multivariate and time-stratified (am/pm) logistic regression models. As for the multiple adjustments, we further included additional variables such as physical activity, BMI, alcohol consumption, and LDL cholesterol, which were not included in the principal analyses.

## Results

A total of 543 participants participated in the study, but 34 (6.3%) had missing information for hs-cTnT and one had an hs-cTnT value >35 ng/l (limit for AMI diagnosis) and were therefore excluded. The final analytic sample comprised 508 participants (mean age of 62.9 ± 5.7 years and 55.1% men). The excluded participants did not differ significantly from the main sample in any covariates. The sample is described according to cortisol stress response in Table 1 and according to hs-cTnT categories in Table 2. The prevalence of detectable hs-cTnT was 16.7%. Older and male participants were more likely to have detectable hs-cTnT and higher salivary cortisol responses to stress tasks. hs-cTnT, cortisol response, and CAC appeared to be associated with each other: participants with detectable levels of hs-cTnT were more likely to have higher cortisol responses and CAC (Table 2), and participants having high cortisol responses were more likely to have CAC ≥100 (Table 1). Participants with cortisol responses <4 nmol/l were more likely to be smokers, and those with detectable levels of hs-cTnT had higher IL-6 plasma concentrations (Tables 1 and 2). Baseline cortisol was not associated with hs-cTnT either in bivariate or multivariate models.

**Table 1:** Characteristics of the Study Sample by Categories of Cortisol Response to Laboratory Standard Mental Stress Tasks

Factor and Category	Salivary Cortisol Response		p Value∗
	<4 nmol/l (n = 459)	≥4 nmol/l (n = 35)	
Age, yrs	62.8 ± 5.6	64.7 ± 6.4	0.055
Male	54.5	71.4	0.056
Laboratory session in the morning	39.2	41.2	0.823
Current smoker	5.5	0.0	<0.001
Latest grade of employment			0.071
Higher	38.1	51.4	
Intermediate	39.4	37.1	
Lower	22.4	11.4	
Alcohol consumption			0.943
No alcohol	15.5	17.1	
Below recommended levels	71.2	68.6	
Above recommended levels	13.3	14.3	
Hours of physical activity per week			0.727
<1 h	23.3	17.1	
1–4 h	32.4	42.9	
5–7 h	23.3	14.3	
>7 h	21.0	25.7	
Body mass index, kg/m^2^	25.8 ± 3.8	25.8 ± 4.4	0.931
Systolic blood pressure, mm Hg	128.9 ± 15.6	130.2 ± 15.5	0.628
Diastolic blood pressure, mm Hg	69.7 ± 8.6	70.6 ± 9.8	0.534
Glycosylated hemoglobin, %	5.4 ± 0.4	5.4 ± 0.4	0.984
Triglycerides, g/l	1.2 ± 0.7	1.1 ± 0.7	0.480†
HDL, mmol/l	1.7 ± 0.5	1.6 ± 0.4	0.527
LDL, mmol/l	3.0 ± 0.9	3.1 ± 0.9	0.628
Total cholesterol, mmol/l	5.3 ± 0.9	5.3 ± 0.9	0.977
C-reactive protein at baseline, mg/l	1.0 ± 1.4	1.1 ± 1.0	0.780†
IL-6 at baseline, pg/ml	1.3 ± 0.8	1.6 ± 0.9	0.716
Agatston coronary calcium score			0.037
None	44.7	31.4	
<100	32.5	28.6	
<400	14.2	25.7	
≥400	8.7	14.3	
Agatston coronary calcium score >100	22.9	40.0	0.025
hs-cTnT detectable (>3 ng/l)	14.8	40.0	<0.001

Values are mean ± SD or %.

HDL = high-density lipoprotein; hs-cTnT = high-sensitivity cardiac troponin T; IL = interleukin; LDL = low-density lipoprotein.

∗ p values were computed using univariate logistic regression (Wald test), unless otherwise specified. Ordered categorical variables such as latest grade of employment, alcohol consumption, hours of physical activity per week, and Agatston coronary calcium score were treated as linear.

† Two-sample Wilcoxon rank-sum (Mann-Whitney) test.

**Table 2:** Characteristics of the Study Sample by Categories of hs-cTnT Plasma Concentration

Factor and Category	hs-cTnT		p Value∗
	Undetectable (n = 423)	Detectable (n = 85)	
hs-cTnT, ng/l	—	5.6 ± 3.9	
Age, yrs	62.1 ± 5.1	67.2 ± 6.4	<0.001
Male	51.1	75.3	<0.001
Lab session in the morning	39.8	35.9	0.522
Current smoker	5.4	3.5	0.470
Latest grade of employment			0.190
Higher	40.2	31.8	
Intermediate	38.5	43.5	
Lower	21.3	24.7	
Alcohol consumption			0.201
No alcohol	15.1	20.0	
Below recommended levels	70.9	69.4	
Above recommended levels	14.0	10.6	
Hours of physical activity per week			0.481
<1 h	23.1	22.6	
1–4 h	33.0	32.1	
5–7 h	23.8	17.9	
>7 h	20.2	27.4	
Body mass index, kg/m^2^	25.8 ± 3.9	26.2 ± 3.8	0.436
Systolic blood pressure, mm Hg	128.5 ± 15.3	131.8 ± 17.4	0.082
Diastolic blood pressure, mm Hg	69.5 ± 8.5	70.7 ± 9.7	0.262
Glycosylated hemoglobin, %	5.5 ± 0.4	5.5 ± 0.8	0.516
Triglycerides, g/l	1.2 ± 0.8	1.1 ± 0.7	0.059†
HDL, mmol/l	1.7 ± 0.5	1.6 ± 0.4	0.293
LDL, mmol/l	3 ± 0.8	2.9 ± 0.9	0.243
Total cholesterol, mmol/l	5.3 ± 0.9	5.1 ± 1.0	0.024
Salivary cortisol at baseline, nmol/l	6.5 ± 4.4	7 ± 4.2	0.321
Salivary cortisol response, nmol/l	0.3 ± 2.9	1.7 ± 5.5	0.004
Salivary cortisol response ≥4 nmol/l	5.1	17.1	<0.001
C-reactive protein at baseline, mg/l	1.0 ± 2.7	1.2 ± 3.2	0.101†
IL-6 at baseline, pg/ml	1.3 ± 0.8	1.6 ± 0.9	0.003
Agatston coronary calcium score			<0.001
None	47.3	25.9	
<100	32.9	28.2	
<400	13.2	22.4	
≥400	6.6	23.5	
Agatston coronary calcium score >100	19.9	45.9	<0.001

Values are mean ± SD or %.

Abbreviations as in Table 1.

∗ p values were computed using univariate logistic regression (Wald test), unless otherwise specified. Ordered categorical variables such as latest grade of employment, alcohol consumption, hours of physical activity per week, and Agatston coronary calcium score were treated as linear.

† Two-sample Wilcoxon rank-sum (Mann-Whitney) test.

Table 3 shows the results of multiple logistic regression models. We found a robust association between cortisol response and detectable hs-cTnT (odds ratio [OR]: 3.83; 95% confidence interval [CI]: 1.86 to 7.90; p < 0.001). After adjustment for demographic variables and cardiovascular risk factors, the association between cortisol response and detectable hs-cTnT did not differ from that in the unadjusted analysis. After further adjustments for CRP and IL-6, the association remained (OR: 3.98; 95% CI: 1.60 to 9.92; p = 0.003). The adjustment for CAC score as a binary variable did not change the effect estimates. In sensitivity analyses, the results remained unchanged after further adjustment for physical activity, BMI, alcohol consumption, and LDL cholesterol (data not shown). We also performed sensitivity analyses using different approaches to quantify the cortisol stress response, although the same pattern of results emerged. For example, after treating the stress response as a linear proportional change in cortisol, the fully adjusted OR for hs-cTnT was 1.38 (95% CI: 1.10 to 1.73; p = 0.005) for each unit increase in change.

**Table 3:** Multiple Logistic Regression Models for the Association Between Salivary Cortisol Response to Standard Laboratory Mental Stress Tasks (Binary Exposure) and Plasma Detectable hs-cTnT (Binary Outcome)

Model for Detectable hs-cTnT (Outcome)	OR for Cortisol Response (Exposure)	p Value	95% CI
1. Crude association	3.83	<0.001	1.86–7.90
2. Adjusted for baseline salivary cortisol (pre-task) and time of the session (am or pm)	3.68	0.001	1.75–7.76
3. With further adjustment for age and sex	2.75	0.021	1.16–6.49
4. With further adjustment for latest grade of employment and smoking	2.95	0.014	1.25–7.00
5. With further adjustment for systolic blood pressure, total cholesterol/HDL ratio, and glycosylated hemoglobin	3.63	0.005	1.49–8.83
6. With further adjustment for CRP and IL-6	3.98	0.003	1.60–9.92
7a. With further adjustment for coronary calcification score, treated as a binary variable with cutoff at 0	3.98	0.003	1.59–9.95
7b. Same as model 7a but with cutoff set at 100	3.97	0.003	1.59–9.91
7c. Model 6 was restricted to participants without coronary calcification (CAC score 0, n = 222)	4.77	0.025	1.22–18.72
7d. Model 6 was restricted to participants with coronary calcification (CAC score >0, n = 286), for which score it was further adjusted using a log-linear variable	7.39	0.001	2.22–24.64

CAC = coronary artery calcification; CRP = C-reactive protein; OR = odds ratio; other abbreviations as in Table 1.

When we restricted the analysis to participants without detectable coronary calcification, the evidence of association remained despite the 40% drop in sample size (n = 222; OR: 4.77; 95% CI: 1.22 to 18.72; p = 0.025). When the analysis was restricted to participants with coronary calcification and adjusted for Agatston CAC score as a log-linear variable, the association between cortisol stress responses and cTnT concentration remained (n = 286; OR: 7.39; 95% CI: 2.22 to 24.64; p = 0.001) (Table 3).

## Discussion

Our results suggest that in healthy participants with no history of CVD, a heightened cortisol response to mental stress is associated with detectable concentrations of circulating cTnT when measured using a high-sensitivity assay. These findings add to previous data from our laboratory that demonstrate an association between cortisol reactivity and CAC 12, 13. However, importantly, although this study confirms the findings from other work that stress responsivity is associated with increased CAC (12), the association between cortisol stress responses and circulating cTnT was independent of CAC, a reliable indicator of subclinical coronary atherosclerosis. Therefore, cortisol may be involved in structural and functional adaptations to the heart as well as in the atherosclerotic process.

The prevalence of detectable hs-cTnT in our British sample was 16.7%, which is similar to levels reported (15.7%) in a nationally representative CVD-free population sample in the United States (20). To our knowledge, this is the first study to examine the association between mental stress and cTnT in humans. Our data are consistent with research on stress-induced Takotsubo cardiomyopathy, in which emotional stress increases troponin and cortisol levels in the absence of coronary artery disease 31, 32. Our data also agree with those of Caligiuri et al. (33), who showed that mental stress in laboratory animals increases troponin levels. Although stress responses seem to be positively correlated with troponin, evidence for a direct or indirect glucocorticoid modulation of troponin is still scarce. The troponin gene contains a transcription factor binding site 34, 35, previously shown to bind the activated glucocorticoid receptor (36). Despite the presence of this binding site, the effect of glucocorticoids on troponin levels does not seem to be via direct modulation of gene expression 37, 38, 39, 40, 41.

Cortisol has a strong diurnal pattern that can cause difficulties in the interpretation of the data; we dealt with this issue by considering stress response as the difference between the post-test and pre-test measurements and by adjusting for cortisol baseline level and day and time of testing at the data analysis stage. We also performed sensitivity analyses using different approaches to quantify the cortisol stress response, although the same pattern of results emerged. It is possible that the cortisol response to stress contributes to the increased levels of troponin T observed here by several indirect mechanisms. First, oxidative stress could play a role in cortisol-induced troponin release 42, 43. Supporting this hypothesis, the temporal change in serum troponin levels matches the increase in the concentration of myocardial malondialdehyde, a marker of free radical lipid peroxidation (44). Second, stress-induced cortisol levels could increase troponin levels by modulating ion channels. For example, dexamethasone, a synthetic glucocorticoid, significantly increases the L-type Ca^2+^ currents in neonatal rat cardiomyocytes (45) and accelerates myocyte spontaneous contractions (46). Finally, cortisol responses to stress could induce troponin levels by potentiation of adrenergic signaling. Corticosteroids potentiate adrenergic signaling and increase muscle contraction and cardiomyocyte hypertrophy (47). Corticosteroid-potentiated adrenergic signaling increases mineralocorticoid and glucocorticoid receptor expression and function in cardiomyocytes (47).

Detectable hs-cTnT is associated with noncardiac conditions such as severe renal disease 48, 49, and theoretically, our results could be due to confounding if patients with renal disease are more likely to test positive on mental stress tests. However, it is unlikely that this mechanism underlies our results because the study participants were free from any chronic conditions at the time of testing, as explained in the Methods section.

The effect of stress-related cortisol release on cardiomyocytes may be mediated by atherosclerosis and consequent ischemia, although the adjustments for CAC, which is a recognized index of atherosclerosis, did not attenuate the findings. CAC and hs-cTnT do not appear to lie on the same causal pathway because their effect was not diminished by mutual adjustment, despite being correlated with each other. Thus, cortisol might have acted through indirect effects as described previously.

Noncalcified coronary plaques are not detectable using cardiac computed tomography, and that may partly explain why CAC did not attenuate the association between cortisol and hs-cTnT. However, there is a direct relationship between the number of calcified plaques present and total plaque burden, and CAC correlates highly with the severity of coronary artery disease; therefore, the absence of calcification implies that there is probably little significant coronary artery disease (50). On the other hand, it has been argued that raised troponin T levels may be due to occult or undetected plaque rupture (51), and it is known that plaque rupture is a relatively common event that is usually not followed by an acute cardiac event (52). This process may have operated in our patients with minimal CAC score levels and detectable hs-cTnT.

A single measure of plasma hs-cTnT concentration cannot be regarded as a robust test if it is not stable in time (i.e., if it shows remarkable intraindividual short-term variation). However, the results from the ARIC (Atherosclerosis Risk in Communities) study showed that hs-cTnT intraindividual variability over 6 weeks is almost null, with a correlation coefficient of 0.94 (53). Thus, although our study collected hs-cTnT after a brief and moderately stressful behavioral challenge, it is improbable that troponin T was released in response to this task. To the contrary, we hypothesized that high cortisol responders are individuals who are hyperreactive to mental stress in everyday life. If these responses are elicited on a regular basis over extended periods of time, they might lead to chronic elevations in hs-cTnT concentrations.

This was a cross-sectional study, and therefore, we cannot determine the causal sequence. Heightened cortisol stress responsivity may contribute to early signs of CVD, or people at an early stage of cardiac disease may be more prone to disturbed stress responses. In fact, cardiac troponins are the most sensitive and specific biochemical markers of myocardial damage (54), but their elevation can be due to a variety of reasons such as pericarditis, myocarditis, pulmonary embolism, and others (55). However, it is unlikely that the undetected presence of those conditions can explain our findings because no participants reported any symptoms or signs of cardiac disease; had any previous diagnosis of or treatment for hypertension, inflammatory disease, or allergies; and did not show any electrocardiographic indications of congenital heart disease on tests carried out over more than 20 years in the Whitehall II study. Moreover, we found a strong association in people with and without coronary calcification, which is consistent with another study showing that hs-cTnT is predictive of CVD in healthy people and of secondary events in patients with CVD (56). It is interesting to note that detectable levels of hs-cTnT were associated with salivary cortisol response to stress test but not with its baseline levels (pre-test), corroborating its relationship with stress-induced neuroendocrine dysregulation.

## Conclusions

Heightened cortisol responses to mental stress were associated with detectable levels of cTnT using high-sensitivity assays in the plasma of healthy people. Heightened stress-induced cortisol release may increase the risk of CHD through several pathways, including atherosclerotic processes, or other indirect effects on the cardiomyocytes. Further research is needed to understand the role of psychosocial stress in the pathophysiology of cardiac cell damage.
